# Postnatally-transmitted HIV-1 variants are efficient at dendritic cell trans-infection and sensitive to autologous and heterologous neutralization

**DOI:** 10.1186/1742-4690-9-S2-P148

**Published:** 2012-09-13

**Authors:** GG Fouda, T Mahlokozera, K Rizzolo, J Salazar-Gonzalez, M Salazar, G Learn, S Barotra, M Sekaran, E Russell, F Jaeger, F Cai, F Gao, B Hahn, R Swanstrom, S Meshnick, V Mwapasa, L Kalilani, S Fiscus, D Montefiori, B Haynes, J Kwiek, M Alam, S Permar

**Affiliations:** 1 Duke Human Vaccine Institute, Durham, NC, USA; 2 Beth Israel Deaconess Medical Center, Boston, MA, USA; 3 University of Alabama at Birmingham, Birmingham, AL, USA; 4 Ohio State University, Columbus, OH, USA; 5 University of North Carolina, Chapel Hill, NC, USA; 6 University of Pennsylvania, Philadelphia, PA, USA; 7 University of Malawi, Blantyre, Malawi

## Background

Postnatal transmission via breastfeeding is a leading cause of infant HIV infection in the developing world. However, only a small minority of breastfed infants born to HIV-infected women become infected. As a genetic bottleneck severely restricts the number of postnatally-transmitted variants, genetic or phenotypic differences in the virus Envelope (Env) may play a role in its ability to breech the mucosal barrier in the infant gastrointestinal tract.

## Methods

We examined the biologic properties of HIV Env pseudoviruses cloned from breast milk of postnatally-transmitting mothers (n=14 viruses), clinically-matched nontansmitting mothers (n=16 viruses), and early viruses from postnatally-infected infants (n = 6).

## Results

There was no difference in epithelial cell attachment, internalization, or gp120 interaction with the putative HIV epithelial cell receptor, galactosylceramide, between milk HIV Env variants from transmitting and nontransmitting mothers. Similarly, there was no difference in the efficiency of milk Env variants to bind to monocyte-derived dendritic cells (DC). However, there was trend towards more efficient DC-mediated trans-infection of CD4-expressing target cells by milk Env variants of transmitting women compared to those of non-transmitting women (p = 0.06). Moreover, early infant Env variants were more efficiently transferred from DC than milk variants of nontransmitting women (p = 0.0009). The high-efficiency DC trans-infection was not attributable to higher infectivity or fusion efficiency of the postnatally-transmitted viruses. Infant Env variants were more sensitive to neutralization by broadly- neutralizing antibodies (HIVIG-C: p=0.02, PG-9: p=0.04, and VRC01: p=0.02) than Env variants from milk of nontransmitting women. In addition, Env variants from transmitting and nontransmitting mothers were equally-sensitive to neutralization by autologous plasma.

## Conclusion

While resistance to broadly-neutralizing antibodies does not appear to be a defining feature of postnatally-transmitted Env variants, efficient HIV Env co-interaction with DCs and CD4-expressing target cells may be required for postnatal HIV transmission via breastfeeding.

